# Editorial: Overcoming resistance to immune checkpoint inhibitors: mechanisms and strategies in cancer therapy

**DOI:** 10.3389/fimmu.2026.1864583

**Published:** 2026-05-06

**Authors:** Margherita Piccinelli, Elio Gregory Pizzutilo, Maria Gemelli, Dhifaf Sarhan, Stefano Cavalieri

**Affiliations:** 1 Head and Neck Medical Oncology Department, Fondazione IRCCS Istituto Nazionale dei Tumori, Milan, Italy; 2 Niguarda Cancer Center, Grande Ospedale Metropolitano Niguarda, Milan, Italy; 3 IRCCS Multimedica, Sesto San Giovanni (MI), Milan, Italy; 4 Division of Pathology, Department of Laboratory Medicine, Karolinska Institutet (KI), Stockholm, Sweden; 5 Department of Oncology and Hemato-oncology, University of Milan, Milan, Italy

**Keywords:** combination immunotherapy, immune checkpoint inhibitors, immunotherapy resistance, predictive biomarkers, tumor microenvironment

Immune checkpoint inhibitors (ICIs) are monoclonal antibodies designed to enhance antitumor immunity by targeting negative regulatory pathways, most notably CTLA-4 and PD-1/PD-L1, which tumors exploit to evade immune surveillance. By blocking these inhibitory signals, ICIs can reactivate T-cell–mediated antitumor responses and promote tumor cell killing.

This Research Topic explores the major biological and molecular mechanisms underlying resistance to immunotherapy, their clinical implications, and potential innovative therapeutic strategies.

ICIs have redefined the management of multiple malignancies and, in several settings, have significantly improved patient prognosis. They are now increasingly incorporated into multimodal cancer treatment strategies alongside chemotherapy, radiotherapy, and surgery.

Nevertheless, a substantial proportion of patients exhibit primary resistance from the outset or eventually develop acquired resistance after an initial response. In fact, ICIs display considerable variability in outcomes, with approximately 20–40% of patients achieving durable responses to PD-1/PD-L1 monotherapy and combination anti CTLA-4/PD-1 therapy, still leaving more than half of patients without durable benefit.

In this context, the case reported by Yang et al. provides a real-world example of an innovative later-line approach in advanced Non-Small-Cell-Lung Cancer (NSCLC), in which the bispecific PD-1/CTLA-4 inhibitor *cadonilimab* was combined with the anti-angiogenic agent *anlotinib* and local radiotherapy, resulting in 11 months of progression-free survival. A notable result in this context, exceeding the 6 months reported in a previous case.

Wang et al. provided a comprehensive review that explains the complex mechanisms underlying resistance to PD-1/CTLA-4 blockade and surveys emerging strategies to overcome them. It highlights three main determinants of resistance: remodelling of the tumor microenvironment, tumor-intrinsic immune escape mechanisms, and immune cell dysfunction.

The tumor microenvironment (TME) plays a critical role in guiding immune intervention and developing innovative therapeutic strategies to target different tumor types. The immunological state of TME can be broadly classified as “*hot*” or “*cold*”.

*Hot tumors*, such as melanoma or NSCLC, are highly inflamed and usually characterized by abundant *intratumoral* T-cell infiltration and increased expression of inflammatory markers. Typically, they are associated with a *higher* Tumor Mutational Burden (TMB) leading to an enhanced neoantigen presentation and a better response to ICIs. While TMB has emerged as a potential predictor of response, its predictive value is inconsistent across tumor types, underscoring the need for more comprehensive biomarkers integrating tumor immunogenicity and microenvironmental features. Lee et al. provided an insightful review of virus-associated cancer and discusses how *viral-driven* cancers represent a distinct immunologic setting. In this context, viral antigens, tumor immunogenicity, and tumor microenvironment interact to influence both sensitivity and resistance to immunotherapy. Notably, *virus-positive* Merkel Cell Polyomavirus (MCC), that has low-moderate TMB, is yet reported to have high objective response rates to ICB. In HPV-positive head and neck squamous cell carcinoma (HNSCC), particularly integration-negative tumors, response to ICB seems to depend not only on viral antigenicity, but also on the composition and functional state of the tumor microenvironment, which may represent an even stronger determinant of treatment outcome. The review suggests that future efforts to overcome resistance in viral-driven cancers may rely on vaccine-based approaches, adoptive cell therapies and biomarker-informed strategies supported by deeper multi-omics characterization of the TME.

*Cold tumors* are characterized by poor immune infiltration, lower TMB and PD-L1 expression. Hu et al. reviewed the role of PD-1/PD-L1 immunotherapy in Prostate Cancer (PCa) explaining how, in this setting, the limited efficacy of PD-1/PD-L1 blockade and highlights the imperative to delineate PCa’s distinct immune evasion mechanism. Notably, in PCa, epigenetic modifiers and signal transducers can regulate the expression of PD-1/PD-L1 through multi-level pathways, shape the TME and promote tumor immune escape. The review also highlights TME remodelling as a potential strategy to overcome resistance.

A third category, based on the distinct immune phenotypes of T-cell spatial distribution, comprises the *immune-excluded tumors*, distinguished by CD8+ T-cell sequestration within tumor stroma, forming a “peripheral immune shield”, resulting in poor prognosis and primary resistance to ICIs. Hou, in a comprehensive mini-review, describes how TGF-β-induced MyoCAF are identified as key mediators in driving immune exclusion and resistance to ICB through dual mechanism: promoting fibrotic TME and forming functional alliances with macrophages (TAM). In this paper, T-cell exclusion is evolving as a potential *biomarker* for immunotherapy resistance. While targeting TGF-β or CAFs directly has shown limited inconsistent results, downstream molecules in TGF-activated CAFs emerge as promising therapeutic targets to counteract T-cell exclusion and restore immunotherapy sensitivity.

Among tumor-intrinsic escape mechanisms, defects in antigen presentation emerge as a key determinant of resistance to immunotherapy. Han et al. provided a focused review about the role of β2 microglobulin (β2M) alterations, showing that β2M deficiency or mutation can significantly impair lymphocyte-mediated recognition of cancer cells, promoting tumor immune evasion and resistance to immunotherapy. Despite this, high immunogenic cancer patients with β2M defect can still exhibit durable responses to anti PD-1 therapy, suggesting the involvement of immune cell subsets beyond CD8+ T cells in the responses (*missing self activation*).

A third mechanism of resistance described by Wang et al., involves immune cell dysfunction, with T-cell exhaustion representing one major example. ICIs therapy, in fact, is less effective if T cells are too exhausted to respond. This understanding has prompted combination strategies, such as combining PD-1 blockade with another check-point inhibitor (anti-LAG3 or anti-TIGIT).

Remaily et al. conducted a study to investigate the role of Fcγ receptor binding in modulating IgG clearance in cancer cachexia, with the aim of clarifying a potential mechanism underlying altered ICIs pharmacokinetics and poorer immunotherapy outcomes. The study showed that tumor induced inflammation and cachexia in the Lewis Lung Carcinoma (LLC) model, alters FcγR expression. These findings underline the fact that cancer cachexia is not merely a marker of poor prognosis but may actively influence immunotherapy efficacy through Fcγ receptor–related alterations in antibody clearance.

Although resistance to PD-1 and CTLA-4 blockade remains a major challenge in modern cancer therapy, considerable progress has been made in unravelling its complex biological basis. These advances are now fuelling the development of innovative therapeutic strategies, including next-generation immunotherapies, bispecific antibodies, and rational combinations designed to target both the TME and tumor-intrinsic escape pathways. Within this evolving landscape, the field of predictive biomarkers is rapidly expanding. A valid strategy could be combining a robust algorithm: TMB, key clinical biomarkers and PD-L1. In this context, Vainer et al. provided a detailed overview of PD-L1 assays and their clinical implications. PD-L1 is an important biomarker for immunotherapy selection, approved assays use different antibodies, platforms, and scoring algorithms, which limits assay interchangeability in clinical practice.

Collectively, these studies offer a broader understanding of the biological complexity underlying immunotherapy resistance and lay the groundwork for more precise and mechanism-based therapeutic interventions.

